# Influence of hypoxia on the domiciliation of Mesenchymal Stem Cells after infusion into rats: possibilities of targeting pulmonary artery remodeling via cells therapies?

**DOI:** 10.1186/1465-9921-6-125

**Published:** 2005-10-27

**Authors:** Gaël Y Rochefort, Pascal Vaudin, Nicolas Bonnet, Jean-Christophe Pages, Jorge Domenech, Pierre Charbord, Véronique Eder

**Affiliations:** 1 LABPART-EA3852, IFR135, Université François Rabelais, faculté de Médecine, 10 boulevard Tonnellé 370032 TOURS France; 2 INSERM ESPRI-EA3588, IFR135, Université François Rabelais, faculté de Médecine, 10 boulevard Tonnellé 370032 TOURS France; 3 Virus, pseudo-virus: morphogenése et antigénicité, EA3856, Université François Rabelais, faculté de Médecine, 10 boulevard Tonnellé 370032 TOURS France; 4 Architecture du Tissu Osseux – Exercice Physique, EA 3895, Université d'Orléans- BP6749, 45067 Orléans cedex 2 France

**Keywords:** arteries, hypertension, pulmonary, hypoxia, lung, remodeling, mesenchymal stem cells.

## Abstract

**Background:**

Bone marrow (BM) cells are promising tools for vascular therapies. Here, we focused on the possibility of targeting the hypoxia-induced pulmonary artery hypertension remodeling with systemic delivery of BM-derived mesenchymal stem cells (MSCs) into non-irradiated rats.

**Methods:**

Six-week-old Wistar rats were exposed to 3-week chronic hypoxia leading to pulmonary artery wall remodeling. Domiciliation of adhesive BM-derived CD45^- ^CD73^+ ^CD90^+ ^MSCs was first studied after a single intravenous infusion of Indium-111-labeled MSCs followed by whole body scintigraphies and autoradiographies of different harvested organs. In a second set of experiments, enhanced-GFP labeling allowed to observe distribution at later times using sequential infusions during the 3-week hypoxia exposure.

**Results:**

A 30% pulmonary retention was observed by scintigraphies and no differences were observed in the global repartition between hypoxic and control groups. Intrapulmonary radioactivity repartition was homogenous in both groups, as shown by autoradiographies. BM-derived GFP-labeled MSCs were observed with a global repartition in liver, in spleen, in lung parenchyma and rarely in the adventitial layer of remodeled vessels. Furthermore this global repartition was not modified by hypoxia. Interestingly, these cells displayed *in vivo *bone marrow homing, proving a preservation of their viability and function. Bone marrow homing of GFP-labeled MSCs was increased in the hypoxic group.

**Conclusion:**

Adhesive BM-derived CD45^- ^CD73^+ ^CD90^+ ^MSCs are not integrated in the pulmonary arteries remodeled media after repeated intravenous infusions in contrast to previously described in systemic vascular remodeling or with endothelial progenitor cells infusions.

## Background

Recent studies emphasize on the perspective of cellular therapy by intravenous stem cells infusion. The participation of stem cells in several vascular diseases pathogenesis was first proved with haematopoietic stem cells (HSCs). In this regard, following bone marrow engraftment, HSCs were observed in remodeled vascular wall following graft vasculopathy or arteriosclerosis [1]. When integrated to the vascular wall, HSCs differentiate into mature vascular cells with an endothelial or smooth muscle cells phenotype.

Mesenchymal Stem cells (MSCs) are bone marrow non-haematopoietic stem cells that are multipotent and can differentiate into bone, cartilage and connective tissue cells [2-4]. They also differentiate in smooth muscle fibers and could be preferential candidates for vascular cells therapies [5]. Moreover MSCs present many advantages as facility to culture or to transform genetically [6]. Surprisingly few studies focused on the domiciliation of MSCs after *in vivo *infusion, even though they can be found into different organs after several months in normal animals, proving the *in vivo *infusion possibility without graft rejection [7]. Barbash *et al *recently showed a MSCs domiciliation into myocardial infarct area, however only a poor fraction of the cells engrafts the myocardium after systemic infusion [8].

Sustained pulmonary hypertension is a common complication of chronic hypoxic lung diseases. Hypoxic pulmonary hypertension is characterized by sustained pulmonary vasoconstriction and pulmonary vascular wall remodeling, including media and adventitia hypertrophy, without endothelial cells disruption. Furthermore chronic hypoxia has been shown to induce capillary angiogenesis [9]. Recently the participation of stem cells to hypoxia-induced adventitial remodeling has been observed in chronically hypoxic rat lungs [10]. Our hypothesis was that MSCs could domicile into the pulmonary artery remodeled wall and thus participate to hypoxia-induced structural changes.

We studied, for the first time, the bone marrow derived CD45^- ^CD73^+ ^CD90^+ ^MSCs domiciliation after intravenous infusion in a model of chronically hypoxic rats, which induces pulmonary artery hypertension and vascular remodeling. Firstly, MSCs distribution was studied after a unique infusion of MSCs labeled by Indium-111 oxinate. Secondly, distribution was studied after sequential infusions of MSCs, transduced with the enhanced green fluorescent protein (GFP) gene by viral infection, during the three weeks of hypoxia exposure.

## Methods

### Animals

Six-weeks-old Wistar male rats (n = 26, Harlan) were exposed for 3 weeks to chronic hypoxia in a hypobaric chamber (50 kPa) to lead the development of pulmonary hypertension and were compared to control matched rats (n = 26).

The MSCs engraftment and viability control was performed using 4 hypoxic rats and compared to 4 control rats by a direct *in-vivo *injection of GFP-labeled MSCs into the right lung parenchyma and checked 3 weeks after normoxic or hypoxic condition housing as described below. The early dynamic distribution of infused radiolabeled MSCs was performed using 6 hypoxic rats and compared to 6 control rats. The long-term distribution of infused GFP-labeled MSCs was performed using 6 other hypoxic rats compared to 6 matched control rats. Finally, 5 hypoxic rats and 5 control rats were also sacrificed for DNA extraction and 5 hypoxic rats and 5 control rats were sacrificed for pulmonary enzymatic digestion and culture (see below).

All animal investigations were carried out in accordance with the Guide for the Care and Use of Laboratory Animals published by the US National Institute of Health (NIH Publications N°85-23, revised 1996) and European Directives (86/609/CEE).

### Cell culture

Cell isolation and culture procedures for MSCs have been established and published previously [11,12]. Briefly, femurs were aseptically harvested from 6-weeks-old Wistar rats and the adherent soft tissue was removed. The proximal and distal ends of the femur were excised at a level just into the beginning of the marrow cavity. Whole marrow plugs were obtained by flushing the bone marrow cavity with a 18-gauge needle set with a syringe filled with culture medium composed of Modified Eagle Medium Alpha (α-MEM; Invitrogen) supplemented with 20% fetal calf serum (FCS; Hyclone), with antibiotic solution (penicillin/streptomycin: 1%; Invitrogen) and with antimycotic solution (amphotericin B: 0.01%; Bristol-Myers). The marrow plugs were dispersed to obtain a single cell suspension by sequentially passing the dispersion through 18- and 22-gauge needles. The cells were centrifuged and resuspended with culture medium. After counting in Malassez cells following an acetic acid disruption of red blood cells, nucleated cells were plated at a density of 10^6^/cm^2 ^and incubated at 37°C in a humidified atmosphere of 95% air 5% C0_2_. The first medium change was after 2 days and twice a week thereafter. When these primary MSCs reached 80–90% of confluence, they were trypsinized (trypsin-EDTA, Invitrogen), counted and passaged at a density of 10^4^/cm^2^. For the first study second-passage MSCs were labeled with ^111^In-oxine as described below and infused intravenously. For the second study MSCs were GFF-labeled after viral gene transduction after the first passage and were used as the second-passage.

Adherent second-passage MSCs were analyzed by flow cytometry with a FACSCalibur flow cytometer (Becton-Dickinson) using a 488 nm argon laser. Cells were incubated for 60 minutes at 4°C with phycoerythrin- or fluorescein isothiocyanate-conjugated monoclonal antibodies against rat CD45 (clone OX-1), rat CD73 (clone 5F/B9), and rat CD90 (Clone OX-7; all from Becton Dickinson). Isotype-identical antibodies served as controls. Samples were analyzed by collecting 10,000 events on a FACSCalibur instrument using Cell-Quest^® ^software (Becton-Dickinson).

### Isotopic labeling and Indium-111 labeled MSCs intravenous infusion

The cells were incubated with ^111^In-oxine (37 MBq/10^6 ^cells) and incubated for 60 minutes as previously described [11]. The radiolabeled MSCs were aliquoted at 10^7 ^cells/ml and intravenously infused to hypoxic rats within 1 hour and followed by whole body scintigraphic imaging. Preliminary experiments showed that the viability and growth of these labeled MSCs were not adversely affected by this labeling procedure (data not shown); the level of radioisotope was widely sufficient to produce high quality images taken with a gamma camera and to produce high quality autoradiographic images of organs.

Whole body scintigraphic imaging was performed immediately after infusion and within 15 minutes, 30 minutes, 1 hour, 3 hours, 24 hours and 96 hours thereafter. Planar whole body images were acquired with Helix Elscint scanner (GE Healthcare) using a medium energy collimator. Images were acquired on a 256 × 256 matrix using a window centered at 245 keV. The distance between the chest of animals and the detector was fixed at 65 mm. In analysis of the scintigraphic images, regions of interest (ROIs) were placed over lungs, liver and spleen on anterior incidence, and over kidneys on posterior incidence. The whole body count was determined by the mean counts on both incidences. Total counts in the ROIs were corrected with physical decay of ^111^In and with body count.

After sacrifice lung, liver, heart, spleen, kidneys and bone marrow were harvested. Organs were weighted and assayed for radioactivity using a Muller counter (Ludlum Measurements), after what they were snap-frozen in liquid nitrogen, whereas cytospins of bone marrow were realized. Sample sections (15 μm) and bone marrow cytospins were exposed to a photographic film within 24–96 hours and autoradiographic films were developed.

### GFP labeling, *in vivo *engraftment and viability controls, and GFP-labeled MSCs intravenous infusions

#### GFP labeling

MSCs were labeled by green fluorescent protein (GFP) after stable viral gene transduction with LNCX-GFP vector. GFP fluorescence from first-passage transduced MSCs was checked by flow cytometry. Non-specific fluorescence was determined using MSCs that were not transduced. GFP-labeling stability was assayed by flow cytometry using tenth-passage GFP-labeled MSCs.

#### In-vivo engraftment and viability controls

Animals were lightly anesthetized and GFP-labeled MSCs were injected, at a dose of 2.10^6 ^cells, through the rib cage, into the right lung lower lobe. After recovering, animals were housed 3 weeks either in normoxic condition, or hypoxic condition. Animals were sacrificed after the 3 weeks and the lung was harvested, snap-frozen in liquid nitrogen. The frozen sample sections (15 μm) were analyzed by tree-dimensional confocal laser microscopy.

#### GFP-labeled MSCs intravenous infusions

Second-passage GFP-labeled MSCs were sequentially infused intravenously at the dose of 10^6 ^MSCs. The first infusion indicated the first day of the 3 weeks chronic hypoxia. Both hypoxic and control rats were infused twice a week during 3 weeks.

After sacrifice lung, liver, heart, spleen, kidneys and bone marrow were harvested. Organs were weighed and snap-frozen in liquid nitrogen. The frozen sample sections (15 μm) of the different organs were analyzed by tree-dimensional confocal laser microscopy. Data was collected with sequential laser excitation to eliminate bleed through and acquired on a 1024 × 1024 matrix using a 110 μm pinhole and an optical section thickness of 0.31 μm. The system was made up of a FV500 confocal microscope (Olympus) using FluoView500 software and a 488 nm argon laser.

The GFP protein was also researched on frozen sections by immunohistochemistry. Sections of harvested organs were incubated with a rabbit polyclonal antibody against GFP (1/200, Santa Cruz Biotechnology) and were revealed either by a conjugated goat anti-rabbit alexa-594 (1/400, Molecular Probes) or by a conjugated goat anti-rabbit horseradish peroxydase (1/400, Biosource).

### Bone marrow homing detection

Cytospins of bone marrow aspirates from control and hypoxic rats were realized 3 days after a unique GFP-labeled MSCs infusion and 3 days after the end of GFP-labeled MSCs infusion during the 3-week hypoxia exposure. The percentage of fluorescent cells was estimated for each rat in five random fields by microscopy using Optimas software (Imasys). Thin slices (12 μm) of frozen bone sections were cut in the metaphysis of tibia from five injected rats. Fluorescence (GFP) was directly observed by confocal microscopy and adipocytes were detected after counterstaining with DAPI (4,6-diamidino-2-phenylindole, AbCys) [13].

### Detection of GFP transgene and protein by PCR and western blotting

After sequential infusions, organs were harvested. From each animal, GFP transgene and protein were assayed by PCR and Western blotting.

#### PCR

Total DNA was extracted using QIAamp DNA Mini Kit (Qiagen, Hilden, Germany) according to the manufacturer's instructions. It was analyzed by PCR for GFP transgene presence using a set of primer generating a 249 bp amplicon: forward, GCGACGTAAACGGCCACAAGTTC and reverse, CGTCCTTGAAGAAGATGGTGCGC. DNA was subjected to PCR for 35 cycles of 94°C for 30 seconds, 58°C for 60 seconds, 72°C for 30 seconds, with a final elongation step of 10 minutes at 72°C.

#### Western blotting

Organs were crushed by Turrax and homogenized with lysis buffer [1% sodium deoxycholate, 0.1% SDS, 1% triton X-100, 10 mM Tris-HCl (pH 8.0), 150 mM NaCl and an inhibitor protease cocktail (chymotrypsin-, thermolysin-, papain-, pronase-, pancreatic extract- and trypsin-inhibitor; Roche)] and centrifuged at 20,000 g for 1 h. After purifying and concentrating small proteins from each sample (Centriprep Centrigugal Devices YM-30MW, Millipore) with a nominal molecular weight limit of 30 kDa, proteins were separated on a SDS/12% polyacrylamide gel and then transferred to a nitrocellulose membrane (Amersham). Blots were blocks for 2 h at room temperature with 5% (w/v) non-ft dried milk in Tris-buffered saline [10 mM Tris-HCl (pH 8.0) and 150 mM NaCl] containing 0.05% Tween 20. The membrane was incubated overnight at 4°C with rabbit polyclonal antibody against GFP (1/400, Santa Cruz Biotechnology). The blot was then incubated with the conjugated goat anti-rabbit horseradish peroxydase (1/1000, Biosource) 2 h at room temperature. Immunoreactive proteins were detected with the ECL Western blotting detection system (Amersham).

### Pulmonary enzymatic digestion

Lung from 5 non-hypoxic and 5 hypoxic MSCs-injected rats were cultured after enzymatic digestion. Briefly, rat lungs were harvested, mechanically dissected and the thin pieces were digested with collagenase (0.5 mg/ml, 1 hour at 37°C, Sigma). After wash, the suspension was passed through a cell strainer to remove undigested block and wash in PBS with FCS (20%, Hyclone). Then, the suspension was incubated in trypsin (30 minutes at 37°C, Invitrogen), wash twice in PBS-FCS, counted, plated and incubated at 37°C in a humidified atmosphere of 95% air 5% C0_2_. The first medium change was after 2 days and twice a week thereafter. The GFP fluorescence was checked after 1 and 2 weeks.

### Statistical analysis

Data are presented as mean +/-SEM with statistical significance tested using the two tailed paired t-test or the Mann-Whitney test.

## Results

### Hypoxia-induced pulmonary arteries remodeling and pulmonary hypertension

The hypoxia-induced pulmonary artery hypertension was checked by echocardiography (data not shown). This is pulmonary artery remodeling model already validated and previously reported by our team [14].

### Mesenchymal stem cells

**Figure 1:**
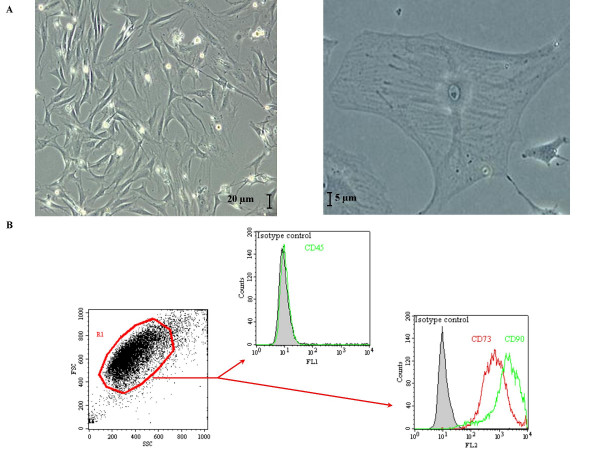
**Mesenchymal stem cells used during this study**. Typical morphological aspects of mesenchymal stem cells observed through culture flask (A). Mesenchymal stem cells expression of CD73 and CD90 antigens was attested by flow cytometry (B).

Cultured bone marrow-derived cells had a typical fibroblast-like morphology and were evenly distributed on the plate after 2 days (fig. 1A). Cells attachment was observed at about 3–4 h and 80–90% of confluence was typically reached by day 6–7. The average cell viability, determined by exclusion of trypan blue, was approximately 90%. CD73 and Thy-1/CD90 were expressed in these MSCs whereas the haematopoietic lineage marker CD45 was not (fig. 1B). These growth patterns and surface markers expression were similar to those of normal rat bone marrow-derived MSCs previously described [12]. Retroviral infection of MSCs had not modified their morphology or viability. The GFP-labeling efficiency was about 98% and the labeling stability was assayed until tenth passage (data not shown).

### Dynamic distribution of radiolabeled-MSCs after a single infusion

**Figure 2:**
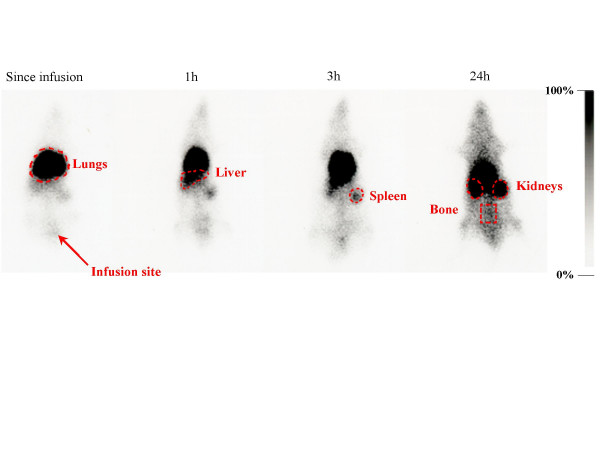
**Early dynamic distribution of mesenchymal stem cells *in vivo***. Sequential whole body scintigraphies after infusion of indium-111 labeled mesenchymal stem cells were acquired from injection up to 96 h. After pulmonary retention, a liver and spleen repartition was observed. A lung domiciliation was indicated by lungs radioactivity stabilization. Bone radioactivity was linked with bone marrow homing after 24 hours.

The distribution of radioactivity after infusion of the radiolabeled-MSCs was imaged from the end of infusion up to 96 h after. This imaging provides an immediate indication of the initial cells distribution. Since radiolabeled-MSCs intravenous infusion, the radioactivity was first observed to accumulate into the lungs, and gradually, the radioactivity was observed in the liver. At 3 h after cell infusion, the radioactivity was observed in the spleen. Kidneys and bone were widely observed at 24 h (fig. 2).

**Figure 3:**
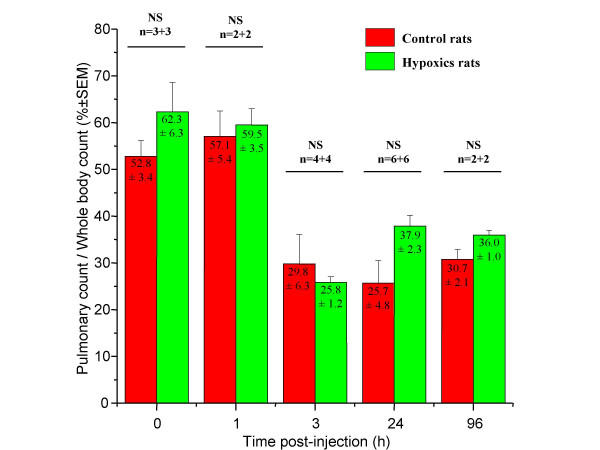
**Pulmonary radioactivity**. Pulmonary repartition was measured *in vivo *from lung region of interest counts on scintigraphies at different times after radiolabeled mesenchymal stem cells infusion. Counts were normalized by whole body counts. After 24 hours, radioactivity was stabilized without differences between control and hypoxic groups.

In order to quantify the distribution of ^111^In, the specific radioactivity of each organ was calculated as a percentage of the total body counts related to the organs region of interest (ROIs) counts. The pulmonary radioactivity was about 50–60% (fig. 3) in both hypoxic and control rats from infusion and at 1 h. This pulmonary radioactivity decreased afterwards and stabilized by about 30% in both groups at 3 h after infusion. No significant difference in lungs ROIs counts was observed between hypoxic rats and control rats (tab. 1).

**Table 1:** Harvested organs radioactivity. The radioactivity repartition in different organs, measured *ex vivo *after animals sacrifice 96 h after radiolabeled mesenchymal stem cells infusion, was normalized by organ weight and by infused activity. The results were corrected by time decay and are presented as mean +/-SEM.

	Control group rats	Hypoxic group rats
Lungs	17.22 % ± 6.92	25.26 % ± 2.78
Liver	41.28 % ± 19,62	29.39 % ± 12.42
Spleen	20.23 % ± 13.59	9.07 % ± 2.83
Kidneys	21.16 % ± 13.01	14.75 % ± 6.93

**Figure 4:**
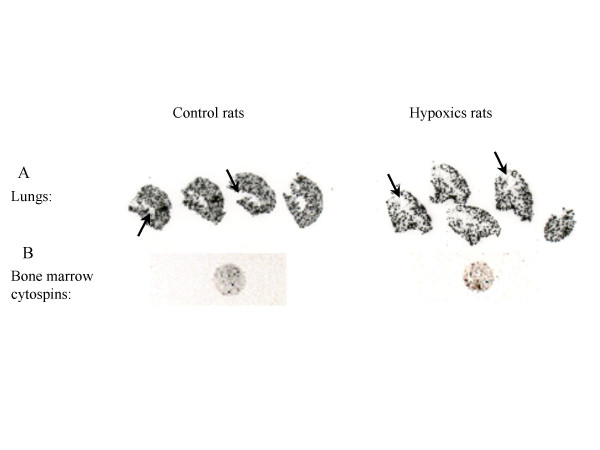
**Autoradiographies**. Autoradiographies of organs frozen sections were realized after animals sacrifice, by 96 h after radiolabeled mesenchymal stem cells infusion. Lung images showed a homogenous repartition and the absence of radioactivity into main arteries that appeared in negative (A, arrows). Lonely signals on bone marrow cytospins confirmed the mesenchymal stem cells homing and excluded free indium bone uptake (B). In all cases no differences in repartition between control and hypoxic groups were observed (see tab. 1).

To observe the distribution of the infused-cells in the lungs, autoradiography of lungs sections were performed (fig. 4A). These films showed homogenous distribution of the radioactivity in both groups. Furthermore, radioactivity was not observed in the lumen of large diameter pulmonary arteries, proving that the infused cells were not agglomerated into the pulmonary vessels lumen.

Bone marrow from radiolabeled-MSCs infused-rats was also harvested and exposed to autoradiographic film. We therefore showed that infused-MSCs homed in bone marrow at 96 h after infusion in both groups (fig. 4B).

### In-vivo engraftment and viability controls

**Figure 5:**
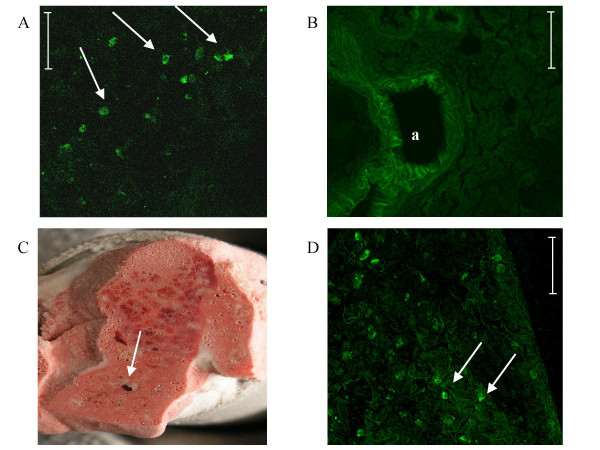
**In-vivo engraftment and viability controls**. GFP signals were researched by confocal microscopy on lungs frozen sections. In a first step, GFP-labeled MSCs were directly injected in *ex-vivo *excised lungs in order to provide positive control (A, arrow) for confocal images interpretation whereas a non-injected freshly harvested lung served as negative control (B). Then, MSCs were directly injected in the right lower lobe of the lung in vivo and rats placed in normoxic or hypoxic conditions for three weeks. Frozen sections of lungs were observed after three weeks in confocal microscopy to provide in vivo positive engraftment and viability controls. Indeed, the injection site was visualized macroscopically (C, arrows) and GFP signals were seen centered on the injection injury (D, arrows). Bar = 50 μm, *a *indicates *artery*.

In order to have positive controls of GFP signals for confocal images interpretation, we first directly injected GFP-labeled cells into a freshly harvested lung (fig. 5A) and compared to non-injected freshly harvested lung (fig. 5B).

To check the *in-vivo *engraftment and viability of the MSCs into lungs, we have directly injected GFP-labeled MSCs into the right lower lobe of the lung and housed animals either in normoxic or hypoxic conditions during 3 weeks. The tolerance of these injections was good and no animals died or showed rejection. From confocal microscopy observation centered on the injection injury (fig. 5C), we observed GFP signals proving the lung engraftment capacity and the viability of the MSCs after 3 weeks (fig. 5D). No difference in the appearance of MSCs was observed between hypoxic and non-hypoxic rats.

### Distribution of GFP-labeled MSCs after sequential infusions

**Figure 6:**
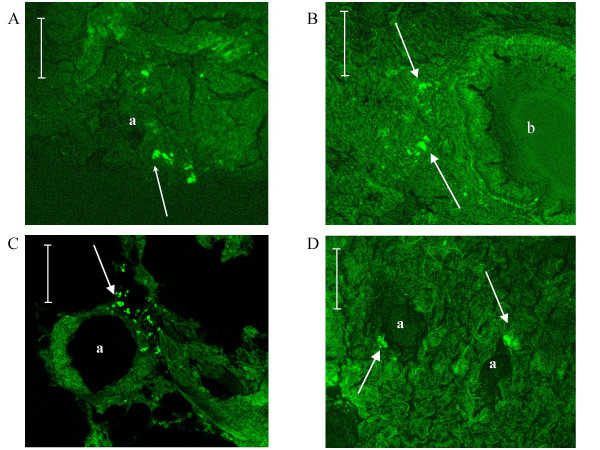
**Mesenchymal stem cell localization in lungs**. GFP-labeled MSCs (arrows) were localized essentially into the pulmonary parenchyma without difference between the non-hypoxic (A) and the hypoxic group (B, C, D). Bar = 50 μm, *a *indicates *artery*, *b *indicates *bronchiole*.

After sequential infusions during the 3-week hypoxia exposure, we examined the harvested lungs sections from control and hypoxic rats. Only few GFP-labeled MSCs were observed per lung sections in both control and hypoxic rats. Moreover when observed, the GFP-labeled MSCs were localized in the lung parenchyma and rarely close to the vascular lumen in both control (fig. 6A) and hypoxic (fig. 6B, 6C, 6D) rats. To localize these cells, we then performed the GFP detection in lungs using immunohistochemistry and peroxydase revelations (data not shown). No signal linked to MSCs localization was observed into the media of pulmonary arteries. Rarely, GFP-labeled MSCs were observed close to the adventitial layer of remodeled vessels. So we confirm the absence of GFP-labeled cells into the remodeled pulmonary arteries.

**Figure 7:**
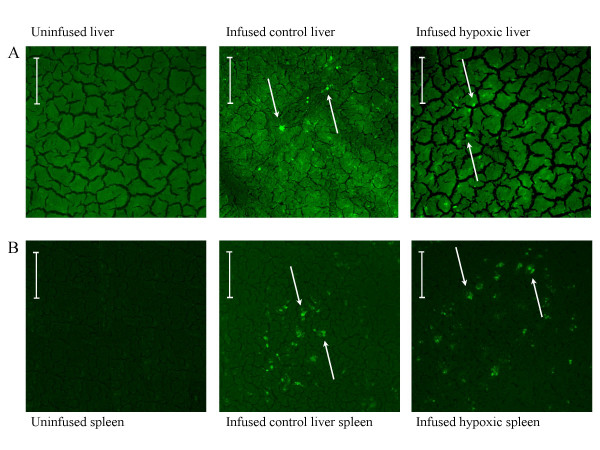
**Mesenchymal stem cell localization in liver and spleen**. GFP signals were observed in liver (A) and spleen (B) from frozen sections after GFP-labeled mesenchymal stem cells infusions observed in confocal microscopy. Hypoxia did not modify their repartition. Arrows refer to GFP signals. Bar = 50 μm.

GFP cells were also and better observed on liver (fig. 7A) and spleen sections (fig. 7B) with the same aspect. No difference in the repartition of GFP-labeled cells was observed in these organs between normoxic and hypoxic groups confirming the absence of pulmonary domiciliation enhanced by hypoxia.

**Figure 8:**
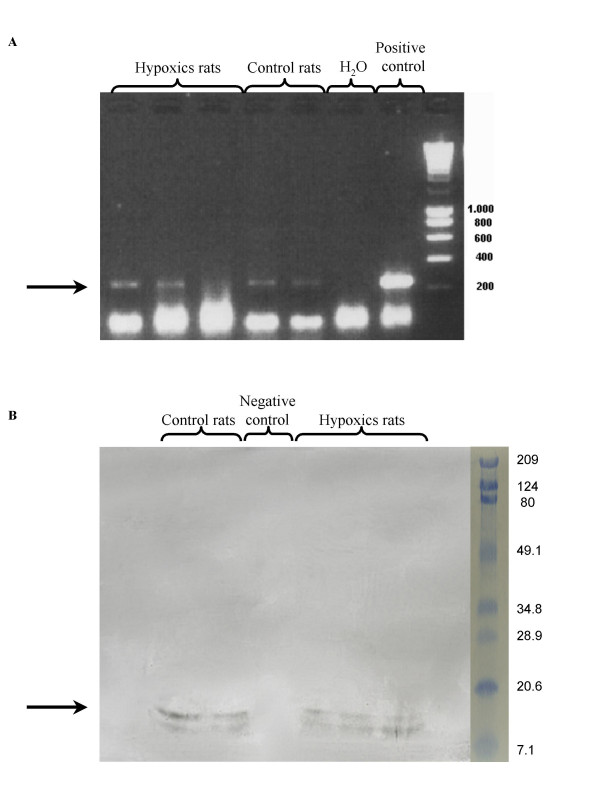
**GFP transgene and protein detection**. After sequential infusions, lungs were harvested. PCR (A) and western blot (B) confirmed presence of GFP transgene and GFP protein in harvested lungs 96 hours after the last infusion in both groups.

The GFP transgene was found in lungs by PCR (fig. 8A) and the GFP protein was recovered in lungs by western blotting (fig. 8B) confirming the presence of GFP-cells into the lungs.

To extract and culture the engrafted GFP-labeled cells from lungs following the same protocol of three-week cell injection and hypoxic exposure, we enzymatically digested lung from 5 control and 5 hypoxic injected rats and cultured. However this experiment failed to obtain cultured GFP-labeled cells suggesting that only few numbers of GFP-labeled cells localized into the lung both in normoxic and hypoxic group.

### Bone marrow homing and engraftment

**Figure 9:**
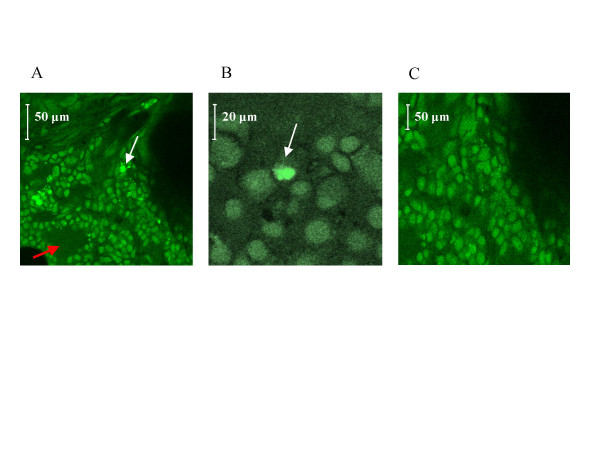
**Bone homing**. After sacrifice, tibia was harvested and slices were cut out from metaphysis and observed by confocal microscopy (A, B). GFP-labeled cells were observed in the same area than adipocytes counterstained by DAPI (white arrows), whereas no green fluorescent was observed in the area of bone marrow (red arrow). A tibia harvested from a non-injected rat was used as control (C).

The fluorescent cell ratio was evaluated on bone marrow cytospins by averaging the results of five views fields for each slide (tab. 2). Compared to a single infusion, we observed an increase of fluorescent cell ratios with sequential infusions (tab. 2) while hypoxia appeared to enhance bone marrow homing. Moreover, on slices of rat tibial bone after GFP-labeled MSCs infusion, we observed fluorescent cells localized between adipocytes (fig. 9A) in contrast to non-infused control rats (fig. 9C). Surprisingly, their appearance in some part looked like the surrounding adipocytes counterstained by DAPI (same size and shape) (fig. 9B). We therefore concluded that MSCs are able to home into bone with preserved viability.

**Table 2:** Bone marrow homing. The evaluation of bone marrow homing, after unique or sequential infusions by evaluation of fluorescent cells ratio on bone marrow cytospins, is presented as mean +/-SEM with statistical significance tested using the Mann-Whitney test.

	Control group rats	Hypoxic group rats	
Unique infusion	2.57 ± 1.48 %	2.76 ± 1.53 %	NS
Sequential infusions	13.01 ± 6.41 %	17.27 ± 5.69 %	p < 0.05
	p < 0.02	p < 0.01	

## Discussion

### Mesenchymal stem cells

Bone marrow comprises both haematopoietic and non-haematopoietic cells among these last mesenchymal stem cells can be found. MSCs in culture can be characterized by their adhesivity, fusiform shape and presence of specific membrane surface antigens. In culture after two passages we showed that more than 90% of collected cells were MSCs. Transduction by GFP did not alter these properties. We did not study the effect of gamma irradiation on the MSCs phenotype. One of the results of our study is that no engraftment intolerance was observed. In accordance with previous studies our results demonstrated that infused MSCs could be found several weeks after infusion. In a precedent study, MSCs were isolated from the receptor organs after *in vivo *infusion and cultured successfully, confirming their viability after domiciliation [15]. Moreover these authors concluded that MSCs could by themselves immuno-privileged. In our study, MSCs morphology and fluorescent labeling were also kept intact and no inflammatory reactions were observed in the surrounding tissue. We then concluded in the absence of graft rejection.

In our study, despite the fact that rats have not been irradiated, we also observed bone marrow homing of MSCs, as previously described for haematopoietic cells after intravenous infusion in immuno-competent animals [16]. This homing could be significantly increased after irradiation [17,18]. In the present study, we observed bone marrow homing of MSCs that was increased by sequential infusions. In bone, some GFP-labeled cells even displayed an adipogenic phenotype, proving *in vivo *their viability. Chondrogenic differentiation of MSCs has already been observed *in vivo *in bone after intravenous infusion in neonatal mice [15]. Nevertheless further studies are required to confirm adipogenic differentiation. Finally, we showed in our hypoxic rat model that 3 weeks after the first intravenous infusion, MSCs remain detectable, viable and functional.

### Pulmonary domiciliation

In our model, adhesive bone marrow derived CD45^- ^CD73^+ ^CD90^+ ^MSCs were localized into the pulmonary parenchyma. After a first phase of pulmonary arteries retention, some MSCs reached the systemic circulation and were distributed mainly in the spleen, and the liver. These cells are essentially observed into the parenchyma of these organs and their presence was confirmed by the detection of the GFP protein in Western Blotting and by detection of the transgene in PCR analysis from lung samples.

This observed global cells distribution is in agreement with previous study [11]. These results leaded some authors to conclude that the pulmonary retention was not specific and without any precise localization neither in the parenchyma nor in the vasculature and to hypothesize that stem cells infusion induces only passive embolism or endothelium adhesion. In our study, we also failed to culture GFP-labeled cells from injected rat lungs suggesting that only few adhesive bone marrow-derived CD45^- ^CD73^+ ^CD90^+ ^MSCs were localized into the pulmonary parenchyma.

Since our autoradiography results showed clearly the absence of radioactivity in the lumen of large diameter pulmonary artery vasculature, we could exclude the idea of a simple intravascular retention of MSCs after infusion. However, we could not exclude MSC localization into the small pulmonary artery walls. Unfortunately, our isotopic labeling did not allow us to analyze the signal observed in peripheral small vessels. Thus, we performed GFP labeling and immunohistochemistry with peroxydase, which confirmed the absence of GFP-labeled cells into the media of small pulmonary artery, as well as into their lumen.

### Effects of hypoxia

Another conclusion of our study is that exposure to chronic hypoxia did not modify the global repartition of MSCs *in vivo*. We demonstrated that after chronic hypoxia, cells were essentially observed into the lung parenchyma. The repartition of MSCs in spleen, liver and bone was unchanged after hypoxic exposure, which argues for a non-specific pulmonary domiciliation.

In hypoxic model, remodeling occurs in the media but also in the adventitial. In a recent study, Hayashida *et al *demonstrated after bone marrow transplantation, that donor's stem cells were present in the remodeled adventitia [10]. However in their studies MSCs do not constitute the major component of the stem cells that can engraft in the bone marrow. So, it is unlikely that stem cells observed by Hayashida *et al *could be mesenchymal stem cells. Moreover, Davie *et al *showed localizations of infused endothelial progenitor in adventitial vaso-vasorum vessels [19]. In another study using the same chronic hypoxic rat model, it has been shown that endothelial progenitor cells could be observed after intravenous infusion in the pulmonary arterioles wall [20]. In this latter study, GFP signal was observed in the parietal wall. However the authors did not focus on what it was observed in control. In our study, fluorescence was observed on the media layer but without difference between hypoxic and control groups and we concluded that artifacts are linked to auto-fluorescence.

Using monocrotaline model of pulmonary hypertension, Zhao *et al *[21] founded local endothelial progenitor cell domiciliation after infusion. However, monocrotaline induces disruption of endothelial layer, which could have a positive impact on this domiciliation contrarily to our hypoxic model without endothelial damaged. We can speculate that in our model, endothelium constitutes a barrier stopping the MSCs incorporation. Our study cannot exclude any participation of mesenchymal stem cells to adventitial remodeling but, in our particular experimental conditions, we did not observed any significant and specific recruitment into pulmonary arterial vasculature after hypoxia exposure. This fact is a major limit to MSCs therapies with intravenous injection.

We showed that MSC bone marrow homing is increased by hypoxia. This interesting finding needs to be confirmed by further studies before speculating a specific effect of hypoxia on MSCs homing, mobilization and migration.

## Conclusion

The major conclusion of our study is that, in our model of hypoxic pulmonary artery remodeling without endothelial disruption, the adhesive bone marrow-derived CD45^- ^CD73^+ ^CD90^+ ^MSCs are not significantly integrated in the parietal wall after repeated intravenous infusion whereas it has been described in systemic vascular remodeling such graft vasculopathy and arteriosclerosis with endothelial progenitor cells.

## List of Abbreviations used

α-MEM modified eagle medium alpha

DAPI 4,6-diamidino-2-phenylindole

DNA deoxyribonucleic acid

ECL enhanced chemiluminescence

FCS fetal calf serum

GFP green fluorescent protein

HSC haematopoietic stem cells

MSC mesenchymal stem cells

PCR polymerase chain reaction

ROI regions of interest

SDF-1 stromal cell derived factor-1

SDS sodium dodecyl sulfate

SEM standard error of mean

## Authors' contributions

GYR conducted the majority of the research experiments. PV and JCP helped with the GFP detection. NB carried out the hypoxic model and helped with GFP detection in bone. JD and PC provided the cell culture equipment. VE conceived the experimental study, participated in its design and coordination and conducted the isotopic experiments. GYR and VE participated in writing and preparation of the manuscript. All authors read and approved the final manuscript.
